# Challenges to decision-making processes in the national HTA agency in Brazil: operational procedures, evidence use and recommendations

**DOI:** 10.1186/s12961-018-0319-8

**Published:** 2018-05-11

**Authors:** Tania Yuka Yuba, Hillegonda Maria Dutilh Novaes, Patrícia Coelho de Soárez

**Affiliations:** Department of Preventive Medicine, University of São Paulo School of Medicine, Av. Dr. Arnaldo, 455, Cerqueira César, São Paulo, SP CEP: 01246903 Brazil

**Keywords:** health technology assessment, health policy, evidence-based policy, evidence-based decisions

## Abstract

**Background:**

The quality of the evidence used in health technology assessment (HTA) agency reports has been considered essential for decision-making processes and their legitimacy. In Brazil, CONITEC is the agency responsible for defining data mandatory for the submission of proposals for the incorporation of new technologies. The objective of this study was to analyse CONITEC recommendation reports, the type of scientific evidence used in them and their compliance with operational procedures.

**Methods:**

This is a descriptive study based on CONITEC official reports from July 2012 through December 2016. Data were collected with a specific extraction form and analysed using descriptive statistics.

**Results:**

We evaluated 199 CONITEC recommendation reports. The annual number of reports increased during the study period. The absolute annual number of new technologies incorporated in 2013 (*n* = 24) was similar to that observed for 2014 (*n* = 24) and 2015 (*n* = 22), decreasing in 2016 (*n* = 13). The type of technology most frequently evaluated was ‘drugs’ (68.3%), followed by ‘procedures’ (20.1%). Overall, 117 (58.8%) reports were internal demands, 75 (37.7%) were external demands and 7 (3.5%) were mixed demands. There were differences between internal and external demands in terms of the evidence used in the reports and the decision regarding the recommendation to incorporate the technologies. Among the internal demands, the recommendation to incorporate the new technology was made for 70.9% of the reports, only 9.6% of which included full HTAs. Among the external demands, the incorporation of the new technology was recommended for 17.3% of the reports, 76.9% of which included full HTAs. Of the 101 reports in which incorporation of the new technology was recommended, 88 (87.1%) did not include a full health economic evaluation and ICER calculation. There are compliance difficulties with the recommendations in the CONITEC internal regulations regarding the type and quality of evidence considered in the analysis of recommendation reports.

**Conclusions:**

The characteristics of the evidence used in recommendation reports and those considered to be mandatory were very different, indicating problems in decision-making processes. There is a need to study, with a broader perspective, the factors that influence the type of evidence used in decision-making processes in order to contribute to the development of better practices and policies.

## Background

Health technology assessment (HTA), a recognised scientific and technological practice, is based on a conceptual framework, uses validated methodologies, is a research topic of interest for academic institutions/research funding agencies, and has social legitimacy. Essentially, HTA proposes the production of knowledge with the potential to contribute explicitly to actions that can positively impact healthcare systems and the health of the population [1–5].

In Brazil, HTA institutionalisation occurred through the creation of structures linked to the Brazilian *Sistema Único de Saúde* (SUS, Unified Healthcare System) [6]. In 2011, the *Comissão Nacional de Incorporação de Tecnologias no SUS* (CONITEC, National Committee for Health Technology Incorporation) was created, with the objective of advising the Ministry of Health (MoH) on policies regarding the incorporation of technologies. The CONITEC internal regulations, established through an ordinance enacted in 2012, state that data related to efficacy, effectiveness, accuracy and safety have to be considered in the proposals submitted demanding the incorporation of new technologies, and that scientific data must be obtained by systematic reviews or other means of scientific knowledge synthesis methodologies, and in accordance with national guidelines for health economic evaluations (HEEs) and budget impact analysis [7, 8]. The CONITEC reports must necessarily take into account the costs and benefits of new technologies compared to those already in use in SUS. As part of a HEE, authors are required to present the incremental cost-effectiveness ratio (ICER) of the technology for comparison with a cost-effectiveness threshold (CET) used in the decision. With the inclusion of HEEs as part of the evidence to be considered, the CONITEC internal regulations follow the recommendations of most HTA agencies in developed countries, in particular the United Kingdom’s National Institute for Health and Clinical Excellence (NICE) [9].

The CONITEC internal regulations also state that additional information should be considered in the preparation of reports, such as the relevance of the technology for SUS health policy priorities, the viability and sustainability of its incorporation into the public health system, the contributions received in the consultations and public hearings, and the degree of innovation and contribution to the technological development of Brazil. The information to be included in the report must be discussed in a session of the Plenary Committee, with the deliberation on the nature of the recommendation in the report being addressed to the Secretary of Science and Technology of the MoH [7, 8].

From an organisational and political point of view, the implementation of CONITEC has shown ongoing development. Its growing technical and political recognition can be measured by the increase in the number of evaluations, the clearer definition of submission and analysis requirements, and the expansion of the committee responsible for the analysis of reports and decision on recommendation. However, certain difficulties have been recognised, such as limited support for the technical staff activities and lack of full transparency in the decision-making processes [10, 11].

The purpose of this study was to analyse the type of scientific information used and the compliance with the operational procedures in the CONITEC recommendation reports. To that end, we characterised the type of technology, the sector demanding incorporation, type of evidence, and the extent to which HEE is used in CONITEC recommendations for incorporation into the SUS.

## Methods

This is a descriptive study based on freely accessible CONITEC official reports published between July 2012 and December 2016. A specific data extraction form was created in order to collect information from each of the reports analysed. The reports were classified according to the type of technology, demanding sector, type of HTA, economic evaluation and ICER calculations/CET comparisons.

The demanding sectors were classified into three groups as internal (the public health sector, the judiciary or other public agents), external (the pharmaceutical industry, civil society, medical equipment companies or the food industry), and mixed (the public health sector and the pharmaceutical industry or civil society).

As detailed in Table 1, the types of studies classified as evidence in the reports were divided into four categories according to the eight criteria of the classification system devised by Merlin et al. [12], namely as full HTA, mini-HTA, rapid review and ‘other’. The minimum criteria of each study were chosen to allow the classification of the reports analysed. A study was classified as a full HTA if it met at least criteria 1, 2, 3, 4 and 6 of the eight Merlin et al. [12] criteria; as a mini-HTA if it met at least criteria 1, 2, 4 and 6; as a rapid review if it met at least criteria 1, 2 and 6; and as ‘other’ if it did not fit into any of the three preceding categories.

**Table 1:** Classification of studies used as evidence in HTA reports according to selected criteria^a^

Study category	Criteria							
	1	2	3	4	5	6	7	8
	Description of the characteristics and current uses of the technology	Evaluation of safety and effectiveness	Cost-effectiveness analysis	Information on costs and financial impact	Organisational considerations	Systematic or systematised review	Critical evaluation of the quality of the evidence	Ethical, social and legal considerations
Full HTA	Always	Always	Always	Always	Optional	Always	Optional	Optional
Mini-HTA	Always	Always	Not performed	Always	Optional	Always	Optional	Optional
Rapid review	Always	Always	Not performed	Optional	Optional	Always	Optional	Optional
Other	Always	Optional	Not performed	Optional	Optional	Optional	Optional	Optional

*HTA* health technology assessment

^a^Based on the classification system devised by Merlin et al. [7]

For the characterisation of the types of HEEs presented in the CONITEC recommendation reports, we employed the classification system devised by Drummond et al. [13], which classifies HEEs as full (cost-effectiveness analysis, cost-utility analysis, cost-benefit analysis or cost-minimisation analysis) or partial (cost description or cost analysis). Budget impact analysis studies were classified as partial economic evaluations.

For the reports that presented a full HEE and ICER, the values were compared with two different concepts of CET:

- The CET adopted by WHO, based on the willingness to pay, suggesting that technologies with ICERs < 1 gross domestic product (GDP) per capita would be highly cost-effective, that those with ICERs of 1–3 GDP per capita would be cost-effective, and that those with ICERs > 3 GDP per capita would not be cost-effective.
- The CET adopted by the University of York Centre for Health Economics, based on the opportunity cost, suggesting that the cost-effective range for Brazil would be US$ 3,210–10,122, which, adjusted by the purchasing power parity established by the World Bank, would, in Brazilian reals (R$), be equivalent to R$ 5,424.90–17,106.18.

For that comparison, all of the ICERs presented in the reports were adjusted to 2016 Brazilian reals, on the basis of the extended consumer price index established by the Brazilian Institute of Geography and Statistics [14]. Data were analysed using descriptive statistics.

## Results

### Characteristics of the CONITEC recommendation reports

CONITEC analysed 541 requests during the period under review (2012–2016). However, we evaluated only the requests that resulted in CONITEC recommendation reports (*n* = 199). Requests that were cancelled for non-compliance with the documentation or at the behest of the requesting party were excluded, as were those that were still under consideration at the end of the study period.

**Fig. 1:**
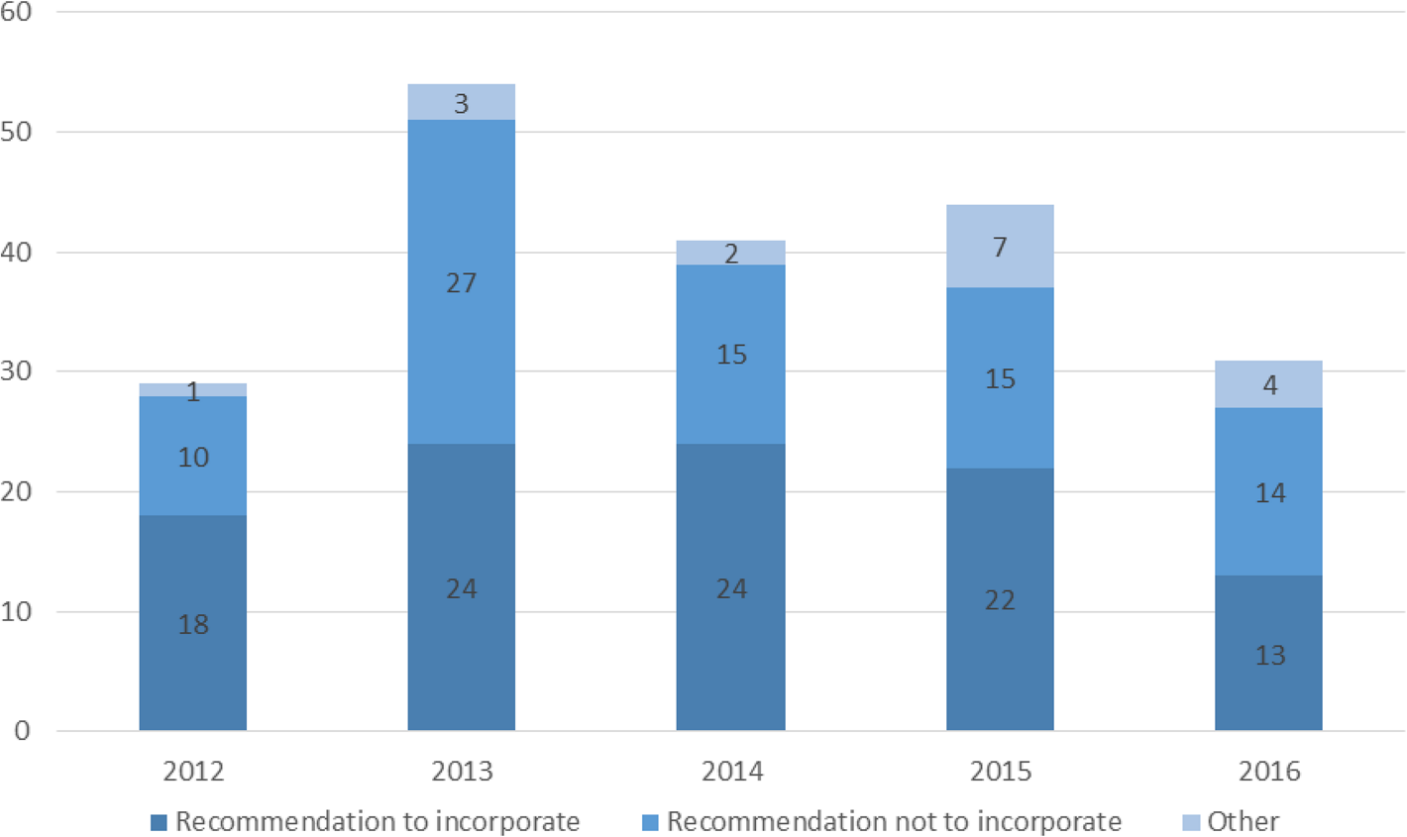
CONITEC reports according to type of recommendation, 2012–2016 (*n* = 199). CONITEC: (Brazilian) *Comissão Nacional de Incorporação de Tecnologias no Sistema Único de Saúde* (CONITEC, National Committee for Health Technology Incorporation); Other: reports recommending broader use or exclusion of the technologies

As shown in Fig. 1, the annual number of reports increased over the course of the study period, peaking in 2013 (*n* = 54). However, the absolute annual number of new technologies recommended to be incorporated in 2013 (*n* = 24) was similar to that observed for 2014 (*n* = 24) and 2015 (*n* = 22), and decreased in 2016 (*n* = 13). Consequently, the proportion of not recommended technologies was higher in 2013 than in 2014, 2015 and 2016 (50.0% vs. 36.6%, 34.1% and 45.2%, respectively).

Throughout the study period, the type of technology most frequently evaluated in the reports was ‘drugs’ (68.3%), followed by ‘procedures’ (20.1%). The categories ‘devices’, ‘dietary supplements’ and ‘vaccines’ accounted each for only a small (11.5%) share (Table 2). The technologies evaluated in the reports (*n* = 199) were mainly related to the group of diseases in Chapter I of the ICD-10 (certain infectious and parasitic diseases; 15.1%, 30/199), followed by Chapter II (neoplasms; 15.1%, 30/199), Chapter X (diseases of the respiratory system; 10.6%, 21/199) and Chapter XIII (diseases of the musculoskeletal system and connective tissue; 10.1%, 20/199) (Table 3).

**Table 2:** CONITEC reports, by type of technology and type of demand, 2012–2016 (*n* = 199)

Variable	2012		2013		2014		2015		2016		Total	
	*n*	%	*n*	%	*n*	%	*n*	%	*n*	%	*n*	%
Type of technology												
Drugs	22	75.9	39	72.2	18	43.9	33	75.0	24	77.4	136	68.3
Procedures	4	13.8	7	13.0	14	34.1	9	20.5	6	19.4	40	20.1
Devices	1	3.4	4	7.4	7	17.1	2	4.5	0	–	14	7.0
Dietary supplements	1	3.4	–	–	2	4.9	–	–	1	3.2	4	2.0
Vaccines	1	3.4	4	7.4	–	–	–	–	0	–	5	2.5
Type of demand												
Internal	14	48.3	25	46.3	34	82.9	30	68.2	14	45.2	117	58.8
Public health sector	13	44.8	23	42.6	32	78.0	29	65.9	13	41.9	110	55.3
Judiciary and public ministry	1	3.4	2	3.7	2	4.9	1	2.3	1	3.2	7	3.5
External	11	37.9	28	51.9	7	17.1	13	29.5	16	51.6	75	37.7
Pharmaceutical industry	11	37.9	28	51.9	4	9.8	12	27.3	9	29	64	32.2
Civil society	–	–	–	–	2	4.9	–	–	4	12.9	6	3.0
Medical device companies	–	–	–	–	–	–	1	2.3	2	6.4	3	1.5
Food industry	–	–	–	–	1	2.4	–	–	1	3.2	2	1.0
Mixed	4	13.8	1	1.9	–	–	1	2.3	1	3.2	7	3.5
Public health sector/the pharmaceutical industry or civil society	4	13.8	1	1.9	–	–	1	2.3	1	3.2	7	3.5
TOTAL	29	100.0	54	100.0	41	100.0	44	100.0	31	100.0	199	100.0

*CONITEC Comissão Nacional de Incorporação de Tecnologias no Sistema Único de Saúde* (National Commission for the Incorporation of Technologies into the Unified Healthcare System)

**Table 3:** CONITEC reports, by ICD-10 chapters and use of health economic evaluation, 2012–2016 (*n* = 199)

ICD-10 chapter	Without HEE	With HEE	Total	Percent
I Certain infectious and parasitic diseases	25	5	30	15.1%
II Neoplasms	14	16	30	15.1%
X Diseases of the respiratory system	9	12	21	10.6%
XIII Diseases of the musculoskeletal system and connective tissue	11	9	20	10.1%
IX Diseases of the circulatory system	2	10	12	6.0%
IV Endocrine, nutritional and metabolic diseases	9	2	11	5.5%
XI Diseases of the digestive system	8	3	11	5.5%
III Diseases of the blood and blood-forming organs and certain disorders involving the immune mechanism	8	2	10	5.0%
XIV Diseases of the genitourinary system	5	5	10	5.0%
XXI Factors influencing health status and contact with health services	7	2	9	4.5%
V Mental and behavioural disorders	6	2	8	4.0%
VI Diseases of the nervous system	3	4	7	3.5%
XII Diseases of the skin and subcutaneous tissue	5	1	6	3.0%
VII Diseases of the eye and adnexa	2	2	4	2.0%
XVII Congenital malformations, deformations and chromosomal abnormalities	2	1	3	1.5%
VIII Diseases of the ear and mastoid process	2		2	1.0%
XVI Certain conditions originating in the perinatal period	1	1	2	1.0%
XIX Injury, poisoning and certain other consequences of external causes	1	1	2	1.0%
XV Pregnancy, childbirth and the puerperium	1		1	0.5%
Total	121	78	199	100.0%

*CONITEC Comissão Nacional de Incorporação de Tecnologias no Sistema Único de Saúde* (National Committee for Health Technology Incorporation), *ICD-10* International Statistical Classification of Diseases and Related Health Problems 10th Revision, *HEE* health economic evaluation

Among the most frequently evaluated, HEEs were mostly conducted in Chapter X (diseases of the respiratory system; 57.1%, 12/21), Chapter II (neoplasms; 53.3%, 16/30) and Chapter XIII (diseases of the musculoskeletal system and connective tissue; 45%, 9/20). There was a balance between reports that presented an HEE and reports without an HEE, except in Chapter I of the ICD-10 (certain infectious and parasitic diseases), where 83.3% (25/30) of the recommendation reports did not present an HEE.

Of the 199 reports evaluated, 117 (58.8%) were internal demands, whereas only 75 (37.7%) were external demands. In the first 2 years (2012 and 2013), external demands outnumbered internal demands, with a reversion in 2014 and 2015, with 34 (82.9%) internal demands out of the 41 demands considered in 2014. In 2016, external demands surpassed internal demands. Among the internal demands, the leading type of demanding party was the public health sector, with the pharmaceutical industry leading the external demands. Figure 1 shows the temporal distribution of the CONITEC reports, according to the recommendation to incorporate or not. In 2012 and 2014, the recommendation was for incorporation in the majority of the reports, although there were no temporal trends in the decisions.

Table 4 shows the main characteristics of the CONITEC recommendation reports, namely the type of evidence, demand, HEE and decision regarding the recommendation to incorporate. Concerning the type of study, 82 (41.2%) of the 199 reports were classified as ‘other’ because they did not meet the criteria to be classified as full HTAs, mini-HTAs or rapid reviews. Of those 82 reports, 64 (78%) only met the ‘description of the characteristics and current uses of the technology’ criterion alone or in combination with the ‘information on costs and financial impact’ criterion. Full HTAs accounted for 78 (39.2%) of the 199 reports, whereas mini-HTAs accounted for 28 (14.1%) and rapid reviews for 11 (5.5%). Of the 199 recommendation reports, 120 (60.3%) presented a systematised or systematic review, and 38 (31.7%) applied a tool for critical evaluation of the quality of the evidence. After 2014, there was an increase in the percentage of reports that critically evaluated the quality of the evidence, especially in 2015 (42.3%) and 2016 (60.0%). The most frequently used tool was the Grading of Recommendations, Assessment, Development and Evaluations (GRADE), with or without another tool (44.7%), followed by the Jadad scale (15.8%), and the Cochrane collaboration tool to assess risk of bias (13.2%). Of the 199 reports, 78 (39.2%) included a full HEE.

**Table 4:** CONITEC reports, by type of recommendation, type of study, type of demand and type of health economic evaluation, 2012–2016 (*n* = 199)

Recommendation, by type of study	Internal request				External request				Mixed request			Total
	Full HEE^a^	CA/CD	BIA	No HEE	Full HEE^a^	CA/CD	BIA	No HEE	Full HEE^a^	BIA	No HEE	
Incorporate, *n* (%)	83 (70.9)				13 (17.3)				5 (71.4)			101 (50.8)
Full HTA	8	–	–	–	10	–	–	–	2	–	–	20
Mini-HTA	–	2	9	–	–	–	1	–	–	–	–	12
Rapid review	–	–	–	2	–	–	–	–	–	–	–	2
Other	–	–	44	18	–	1	1	–	–	2	1	67
Do not incorporate, *n* (%)	17 (14.5)				62 (82.7)				2 (28.6)			81 (40.7)
Full HTA	2	–	–	–	55	–	–	–	1	–	–	58
Mini-HTA	–	1	9	–	–	2	3	–	–	–	–	15
Rapid review	–	–	–	5	–	–	–	1	–	–	–	6
Other	–	–	–	–	–	–	–	1	–	–	1	2
Maintain/expand/exclude, *n* (%)	17 (14.5)				–				–			17 (8.5)
Mini-HTA	–	–	1	–	–	–	–	–	–	–	–	1
Rapid review	–	–	–	3	–	–	–	–	–	–	–	3
Other	–	–	1	12	–	–	–	–	–	–	–	13
Total, *n* (%)	117 (100.0)				75 (100.0)				7 (100.0)			199 (100.0)

*CONITEC Comissão Nacional de Incorporação de Tecnologias no Sistema Único de Saúde* (National Committee for Health Technology Incorporation), *EE* economic evaluation (cost-effectiveness analysis, cost-utility analysis, cost-benefit analysis or cost-minimisation analysis), *CA* cost analysis, *CD* cost description, *BIA* budget impact analysis

^a^Includes a BIA

Among the 117 reports involving internal demands, incorporation of the new technology was recommended in 83 (70.9%). In comparison, incorporation of the new technology was recommended in only 13 (17.3%) of the 75 reports involving external demands. Of the 83 reports involving internal demands that were recommended, 62 (74.7%) were classified as ‘other’, accompanied by the description of the characteristics/current uses of the technology, and included a limited budget impact analysis and no type of economic evaluation. Of the 62 reports involving external demands that were not recommended, 55 (88.7%) were classified as full HTAs and included a full HEE.

### Use of HEEs in the CONITEC reports and decision on incorporation

**Fig. 2:**
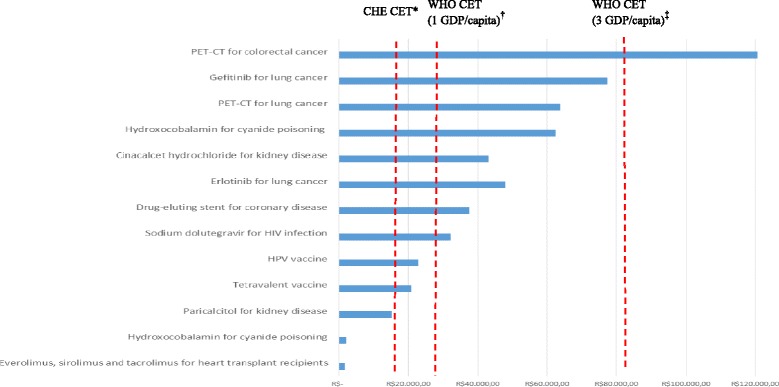
Incremental cost-effectiveness ratios, in 2016 Brazilian reals (R$), for the technologies that gained a CONITEC recommendation for incorporation, 2012–2016 (*n* = 13). *CONITEC Comissão Nacional de Incorporação de Tecnologias no Sistema Único de Saúde* (National Commission for the Incorporation of Technologies into the Unified Healthcare System), *CHE* Centre for Health Economics (University of York), *CET* cost-effectiveness threshold, *WHO* World Health Organization, *GDP* gross domestic product, *PET-CT* positron emission tomography-computed tomography, *HPV* human papillomavirus. *R$ 17,106.18. ^†^R$ 27,229.00. ^‡^R$ 81,687.00

Of the 101 reports in which incorporation of the new technology was recommended, 20 (19.8%) included a full HEE and 13 (12.9%) included a full HEE with ICER calculation. Therefore, 88 (87.1%) of the reports recommended incorporation without a full HEE and ICER calculation. Of the 13 reports including an ICER calculation, 6 compared their results with the CET adopted by WHO. When comparing the ICER values with the WHO CET, we found that 12 of the 13 technologies addressed in those reports would be cost-effective, all 12 showing an ICER < 3 GDP per capita (i.e. < R$ 81,687); of these, 5 technologies showed an ICER ≤ 1 GDP per capita (i.e. R$ 27,229), indicating that they would be highly cost-effective. When comparing the ICER values with the CET adopted by the Centre for Health Economics, we found that only 3 technologies showed ICERs < R$ 17,106.18 (the upper limit of the cost-effective range for Brazil) and would therefore be considered cost-effective (Fig. 2).

Of the 81 reports in which incorporation of the new technology was not recommended, 58 (71.6%) included a full HEE and 45 (55.6%) included a full HEE with ICER calculation. Of the 45 reports including an ICER calculation, 15 compared their results with the CET adopted by WHO. When comparing the ICER values with the WHO CET, we found that 12 of the 15 technologies addressed in those reports would be cost-effective, showing an ICER < 3 GDP per capita (i.e. < R$ 81,687); of these, 7 technologies showed an ICER ≤ 1 GDP per capita (i.e. R$ 27,229), indicating that they would be highly cost-effective. When comparing the ICER values with the CET adopted by the Centre for Health Economics, we found that 5 technologies showed ICERs < R$ 17,106.18 (the upper limit of the cost-effective range for Brazil) and would therefore be considered cost-effective.

## Discussion

The creation of CONITEC in 2011 represented an important step in the institutionalisation of HTA as a specific policy within the overall health policy of the MoH and the SUS. The ongoing activity of CONITEC can be seen in the number of reports produced (199 over the 5 years analysed herein), although no parameters have been established in order to define the number needed to meet the needs of the SUS.

Regarding the type of technology analysed in the CONITEC reports, there was a predominance of drugs (68.3%), as has been the norm for most HTA agencies. Previous studies presented very similar figures of 61.4% [15] and 62.1% [16]. In the period under study, there was also a predominance of internal demands for CONITEC to recommend the incorporation of a given technology, and most of those demands originated in the public health sector, whereas most of the external demands came from the pharmaceutical industry. There were large variations in the proportional distribution of those demands over the years. For example, 82.9% of the reports evaluated in 2014 were generated from internal demands. The significance of such variation remains unclear, and a deeper understanding calls for analyses of the technologies, as well as of their political and economic contexts.

Our findings indicate that there are difficulties related to compliance with the recommendations in the CONITEC internal regulations regarding the type of evidence that should be presented in the recommendation reports. Despite the emphasis that the internal regulations give to economic evaluation as relevant evidence, full HTAs were employed in only 39.2% of the reports. Reports that included an ICER calculation and used an explicit CET to support the recommendation were rare (*n* = 13). The current international debate questions the need for countries to adopt explicit thresholds and recent publications from medium- and low-income countries emphasise the interest in developing thresholds that clearly incorporate budget constraints and opportunity costs in these countries [17–19].

Although we applied a number of criteria for the characterisation of study types lower than that suggested in the International Network of Agencies for Health Technology Assessment classification, we found that nearly half of the reports were classified as ‘other’, as they presented only a description of the characteristics and current uses of the technology, with or without information on its costs and financial impact.

We observed relevant differences between internal and external demands in terms of the evidence used in the reports and the decision regarding the recommendation to incorporate the technology. The higher proportion of recommendations to incorporate among internal demands was also found in a previous study [16]. Among the internal demands, the recommendation to incorporate the new technology was made for 70.9% of the reports, only 9.6% of which included full HTAs. Among those for which the recommendation was not to incorporate, 11.8% included full HTAs. Among the external demands, the incorporation of the new technology was recommended for 17.3% of the reports, and 76.9% of those reports included full HTAs, comparable to the 88.7% observed in the reports for which the recommendation was not to incorporate the new technology.

The two most important findings of the present study are the lack of compliance with what is recommended in the CONITEC internal regulations, in terms of the type and quality of evidence considered in the analysis of the demands for incorporation of new technologies, and the clear difference between the evidence considered in internal demands and in external demands.

The literature on the use of evidence in public policy decision-making provides important indications to understanding the results obtained in our study. The most common explanation for the mismatch between the evidence recommended and the evidence effectively used is the limited scientific knowledge in health and shortage of researchers qualified to produce it, especially in middle- and low-income countries, in addition to the lack of dialogue between researchers and policy-makers. Therefore, there is a need for ongoing investment in research, and for the training of qualified researchers and policy-makers, as well as for the promotion of better communication between the parties. Since 2008, there has been a significant increase in the production of literature in the HTA area in Brazil [20], as well as in the development of courses for training in systematic reviews, technical/scientific reports and guideline dissemination in Brazil. However, all of this is quite recent and there is a need for additional, sustained investment in HTA as a knowledge source and health policy [21, 22].

Nevertheless, this perspective alone does not allow a full understanding of the complex nature of the procedures involved in the use of evidence in decision-making processes and policies. To improve such understanding, there is a need to incorporate studies from the social sciences on policy implementation, showing that they are complex processes in which the initial expectations are always transformed by changing socioeconomic and political conditions, health policies and stakeholder positions [23]. The differences between internal and external demands in terms of the use of evidence indicate that other factors are at play in the development of consensus recommendations. Internal demands may have had greater political legitimacy, having already been analysed and validated in other sectors of the MoH, stimulating recommendation. It is noteworthy that MoH ordinance no. 26, enacted in 2015, redefining the requirements for submission of requests for CONITEC analysis of new technologies to be incorporated, excluded the need to include economic evaluations in the demands [19]. CONITEC is a very recent HTA body, and has contributed with many advances in technology incorporation, but it is still undergoing a process of implementation, with barriers and constraints dependent on a sustainable and adequate relation with health policies for the SUS as a whole.

## Conclusions

An analysis of the changes that CONITEC underwent in the period under study, in terms of the forms of representation of social interests, their representative dynamics and their relations with the MoH, is necessary in order to understand the differences between the evidence recommended and that used in the reports. The deepening of the understanding of the political and institutional factors that affect the use of evidence in decision-making processes, which are not only technical/administrative but eminently political as well as part of the exercise of a democratic deliberative political practice, may allow the identification of the elements required for analyses that are more comprehensive and complex. Such analyses could contribute to the understanding of past and current contexts, as well as to the development of new practices and policies [24, 25].

## Abbreviations

CET: cost-effectiveness threshold
CONITEC: *Comissão Nacional de Incorporação de Tecnologias no SUS* (National Committee for Health Technology Incorporation)
GDP: gross domestic product
HEE: health economic evaluation
HTA: health technology assessment
ICER: incremental cost-effectiveness ratio
MoH: Ministry of Health
SUS: (Brazilian) *Sistema Único de Saúde* (Unified Healthcare System)
